# Intestinal Continuity Alleviates Pediatric Intestinal Failure-Associated Liver Disease

**DOI:** 10.3389/fsurg.2022.881782

**Published:** 2022-05-16

**Authors:** Jinling Wang, Weihui Yan, Lina Lu, Yijing Tao, Liufang Huang, Wei Cai, Ying Wang

**Affiliations:** ^1^ Division of Pediatric Gastroenterology and Nutrition; Xinhua Hospital, School of Medicine, Shanghai Jiao Tong University, Shanghai, China; ^2^ Shanghai Key Laboratory of Pediatric Gastroenterology and Nutrition, Shanghai, China; ^3^ Department of Pediatric Surgery; Xinhua Hospital, School of Medicine, Shanghai Jiao Tong University, Shanghai, China; ^4^ Shanghai Institute for Pediatric Research, Shanghai, China

**Keywords:** intestinal continuity, short bowel syndrome, intestinal failure associated liver disease, anastomosis, growth in intestinal length

## Abstract

**Background:**

Type I short bowel syndrome (SBS) occurs after a critical reduction in the functional gut mass and resection of intestinal continuity after ileostomy or jejunostomy for necrotizing enterocolitis (NEC), intestinal atresia or other causes. SBS is often accompanied with intestinal failure-associated liver disease (IFALD) who requires long-term parenteral nutrition (PN). Our study aimed to observe the effect of intestinal continuity on the hepatic function of pediatric intestinal failure (IF) patients with type I SBS.

**Methods:**

The pre-and post-anastomosis medical records of 35 pediatric patients with type I SBS from April 2013 to April 2019 were reviewed retrospectively. The average growth (cm/month) in the proximal and distal small bowel lengths was calculated as the growth in intestinal length (cm)/the duration (month) from enterostomy to anastomosis. The changes in hepatic function from enterostomy to anastomosis were evaluated by assessment of hepatic function before anastomosis for 6 weeks and after anastomosis for 4 weeks.

**Results:**

The average growth in proximal intestinal length was 9.3 cm/month (±7.2) in neonates and 2.8 cm/month (1.3, 11.9) in infants and children, and in distal intestinal length was 1.5 cm/month (0, 2.7) in neonates and 0.4 cm/month (0, 1.4) in infants and children. The incidence of IFALD was 28.6% 1 month before anastomosis and 20.0% 1 month after anastomosis (*p* < 0.05).

**Conclusion:**

In pediatric type I SBS with IFALD, restoration of intestinal continuity may alleviate liver injury. There was an intestinal compensatory effect on the growth in the intestinal length after resection, and better results were seen in neonates in terms of intestinal length growth.

## Introduction

Intestinal failure (IF) is caused by a constellation of conditions including a reduced absorptive surface area due to surgical or congenital loss of intestinal length, disorders of gastrointestinal motility, and congenital enterocyte defects (1, 2). The most common cause of pediatric intestinal failure is short bowel syndrome (SBS) which occurs as a result of extensive small bowel loss (2). Type I SBS, which leads to a critical reduction in the functional gut mass and resected intestinal continuity after ileostomy or jejunostomy, necessitates long-term parenteral nutrition (PN) to satisfy the body’s nutrient and fluid requirements for growth (2, 3). Intestinal failure-associated liver disease (IFALD) is the most frequent complication of long-term PN in children with SBS. It is regarded as the greatest contributor of morbidity and mortality in pediatric IF cases and is the leading indication for intestinal transplantation (4–8). The causes of IFALD are complex and multifactorial; its risk factors can be divided into two categories: patient-related factors and PN-related factors (9). Patient-dependent risk factors include prematurity, early and recurrent sepsis, small intestinal bacterial overgrowth (SIBO), paucity of enteral nutrition, and compromised bowel anatomy and function after abdominal surgery; and the main factors related to PN which may determine the development of liver injury in children with IF are inappropriate use of lipid emulsions, lack of antioxidants and the presence of phytosterols in the lipid emulsion, micronutrient imbalances and the administration of excessive amounts of glucose, and duration of the infusion period (7–15). However, there are few studies reporting the association between intestinal continuity and IFALD. Therefor the aim of this study was to observe the impact of intestinal continuity on the hepatic function of pediatric IF patients with type I SBS.

## Subjects and Methods

### Patients

A total of 35 patients with type I SBS were admitted to the Division of Pediatric Gastroenterology and Nutrition and Department of Pediatric Surgery, Xinhua Hospital, School of Medicine, Shanghai Jiao Tong University from April 2013 to April 2019. In our study, the diagnosis of SBS was considered when a residual bowel length below a critical value for adequate nutritional supply and required PN for more than 42 days (16, 17). These patients underwent anastomosis and received nutritional support at our hospital. In our center, refeeding of proximal stoma effluent or mush in the distal bowel were encouraged during ileostomy or jejunostomy to stimulate mucosal growth and intestinal adaptation, and prevents atrophy of the distal bowel. Anastomosis was considered to be performed generally in 3 months after ileostomy or jejunostomy, and intestine morphology and function were evaluated through gastrointestinal tractography before anastomosis. The pre-and post-anastomosis medical records of the patients were reviewed retrospectively, including sex, gestational age, birth weight, age at surgery, length of the small intestine in enterostomy and anastomosis, diagnoses, and hepatic function assessment. Ethical approval was obtained from the Research Ethical Committee, Xinhua Hospital, School of Medicine, Shanghai Jiao Tong University, and written informed consent was obtained from the parents of all patients.

### Measurement of Growth of the Small Intestine

The proximal length of the small intestine was measured as the residual intestinal length (cm) from the ligament of Treitz to the stoma, and the distal intestinal length was measured as the residual intestinal length (cm) from the stoma to the ileocecal valve. The length of the small intestine was measured separately at enterostomy and anastomosis, and the growth in the length of the small intestine (cm) was expressed as the difference in the lengths of the small intestine at anastomosis and enterostomy. The average growth (cm/month) in the proximal and distal intestinal lengths was calculated as growth in the length of the small intestine/the duration (month) from enterostomy to anastomosis.

### Hepatic Function

Hepatic function was assessed by the automated colorimetric method (Beckman SYNCHRON LX20 system, Beckman Coulter, CA, USA). The indicators of hepatic function included total bile acids (TBA), alanine aminotransferase (ALT), aspartate aminotransferase (AST), alkaline phosphatase (ALP), γ-glutamyl transferase (GGT), total bilirubin (Tbi), and direct bilirubin (Dbi). The reference ranges were as follows: TBA, 0–10 µmol/L; ALT, 0–75 U/L; AST, 8–38 U/L; ALP, 42–121 U/L; GGT, 16–73 U/L; Tbi, 3–22 µmol/L; and Dbi, 0–5 µmol/L. The hepatic function was assessed before anastomosis for 6 weeks and after anastomosis for 4 weeks to evaluate the changes in hepatic function. The incidence of IFALD was recorded 1 month before and after anastomosis. IFALD was indicated when the serum levels of any three of the seven hepatic function indicators were two times higher than normal without other causes of liver injury including cytomegalovirus infection, viral hepatitis, biliary atresia, choledochal cysts, congenital infections, or metabolic diseases (18).

### Nutrition Support

Nutrition support included enteral nutrition (EN) and parenteral nutrition (PN). Total parenteral nutrition (TPN) was provided during the first few days after ileostomy, jejunostomy, or anastomosis. The “all-in-one” solution contained lipids (Omegavan®, Fresenius Kabi or Lipofundin®, B. Braun Melsungen), amino acids (18AA-11; Treeful, Shanghai, China), a glucose solution, minerals, trace elements (Addamel; Fresenius Kabi, Wuxi, Jiangsu, China), water-soluble vitamins (Soluvit; Fresenius Kabi, Wuxi, Jiangsu, China), and fat-soluble vitamins (Vitalipid; Fresenius Kabi, Wuxi, Jiangsu, China) and was infused continuously via peripherally inserted central catheters (PICCs) or central venous catheters (CVCs) by infusion pumps (SN-1500H, Shenzhen, China). We replaced the medium chain triglyceride/long chain triglyceride (MCT/LCT)-based lipids with fish oil-based lipids (Omegavan; Fresenius Kabi, Jiangsu, China) when the levels of any three of the seven liver indicators were two times higher than normal. Enteral nutrition (breast milk, hydrolyzed formula, or amino acid-based formula) was introduced by pumping for 1–3 h through a nasal tube every 3 h, and advanced gradually based on intestinal tolerance. PN was decreased and weaned off when EN increased sufficiently to sustain adequate growth.

### Statistical Analysis

The results are presented as n (%) for categorical data, mean ± standard deviation for normally distributed data, and median (interquartile range) for other types of data. SPSS 23.0 software for Windows (SPSS Inc., Chicago, IL, USA) was used for statistical analysis. A *p* value <.05 was considered to indicate statistical significance. The growth in the lengths of the proximal and distant intestine and duration from enterostomy to anastomosis were described separately in the population of neonates and infants and children. A paired t-test was performed for normally distributed data, and the Wilcoxon nonparametric test was used for non-normally distributed data. The incidence of IFALD was analyzed using Pearson’s chi-square test.

## Results

Among the 35 pediatric IF patients with type I SBS, there were 19 boys and 16 girls, and 31 patients (89%) had undergone surgery as neonates. The etiologies were distributed across NEC (*n* = 17, 48.6%), intestinal atresia (*n* = 10, 28.5%), Hirschsprung’s disease (HD) (*n* = 3, 8.6%), volvulus (*n* = 2, 5.7%), and miscellaneous (*n* = 3, 8.6%). Thirteen patients (37.1%) changed to fish oil-based lipids for 18 days (9, 32). Two patients (5.7%) died because of infection and liver failure (Table 1).

**Table 1:** Clinical characteristics of the patients

Characteristics	Number
Gender (boy/girl)	19/16
Age at surgery (≤28 d/>28 d)	31/4
Gestational age (d)	232 (212, 258)^a^
Birth weight (g)	2359.5 ± 822.8^b^
Etiology of SBS (%)	
NEC	17, 48.6%
Intestinal Atresia	10, 28.5%
HD	3, 8.6%
Volvulus	2, 5.7%
Miscellaneous	3, 8.6%
Fish oil-based lipids (%)	13, 37.1%
Fish oil-based lipids duration (d)	18 (9, 32)
Death (%)	2, 5.7%

*NEC, necrotizing enterocolitis; HD, Hirschsprung’s disease.*

*^a^* *Median (interquartile range) (all such values).*

*^b^* *Mean ± standard deviation (all such values).*

The results of growth of the proximal and distal small intestine are shown in Table 2. The duration from enterostomy to anastomosis was 5.8 months (±3.6) in neonates and 5.2 months (3.6, 7.3) in infants and children. The average growth in the proximal intestinal length was 9.3 cm/month (±7.2) in neonates and 2.8 cm/month (1.3, 11.9) in infants and children. The average growth in the distal intestinal length was 1.5 cm/month (0, 2.7) in neonates and 0.4 cm/month (0, 1.4) in infants and children.

**Table 2:** Growth in the length of the small intestine

	Neonates (*n* = 31)	Infants or children (*n* = 4)
Proximal length of the small intestine at enterostomy, cm	60.0 (50.0, 80.0)^a^	55.0 (31.3, 75.0)
Distal length of the small intestine at enterostomy, cm	35.0 (4.5, 40.0)	15.5 (0.8, 37.5)
Duration from enterostomy to anastomosis, months	5.8 ± 3.6^b^	5.2 (3.6, 7.3)
Proximal length of the small intestine at anastomosis, cm	94.0 (73.5, 142.0)	74.8 (47.3, 130.4)
Distal length of the small intestine at anastomosis, cm	40.0 (31.5, 83.5)	32.0 (6.1, 101.8)
Average growth in the proximal intestinal length, cm/months	9.3 ± 7.2	2.8 (1.3, 11.9)
Average growth in the distal intestinal length, cm/months	1.5 (0, 2.7)	0.4 (0, 1.4)

*^a^* *Median (interquartile range) (all such values).*

*^b^* *Mean ± standard deviation (all such values).*

**Figure 1:**
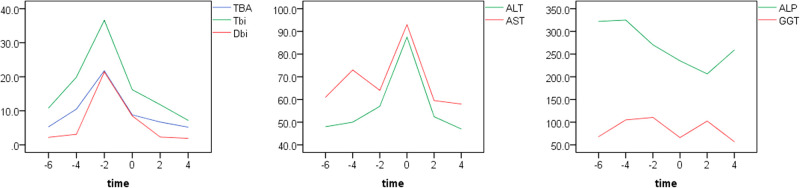
Hepatic function from 6 weeks before anastomosis to 4 weeks after anastomosis. The “zero” on the x-axis represents the time point when anastomosis was performed.

Regarding changes in the hepatic function from enterostomy to anastomosis (Figure 1), there was an obvious increase in TBA, Tbi, Dbi, ALT, and AST before anastomosis and a visible decrease after anastomosis. The results of the changes in the hepatic function before and after anastomosis (2 weeks and 4 weeks before and after) are shown in Table 3. The serum levels of TBA (*p* = 0.028), ALP (*p* = 0.006), and Dbi (*p* = 0.015) 2 weeks before anastomosis were significantly higher than those at 2 weeks after anastomosis, and Tbi (*p* = 0.015) was markedly lower at 4 weeks after anastomosis than before anastomosis. Regarding the hepatic function before anastomosis, Tbi (*p* = 0.000) was significantly higher at 2 weeks than at 4 weeks. As for the hepatic function after anastomosis, Dbi (*p* = 0.046) was significantly higher at 2 weeks than at 4 weeks. The incidence of IFALD 1 month before anastomosis (28.6%) was significantly higher than that at 1 month after anastomosis (20.0%) according to Pearson’s chi-square test (*p* < 0.05).

**Table 3:** Hepatic function.

Hepatic function	Before anastomosis			After anastomosis			*p* ^c^	*p* ^d^
	2 weeks	4 weeks	*p* ^a^	2 weeks	4 weeks	*p* ^b^		
TBA (µmol/L)	21.8 ± 41.3^e^	10.5 (3.0, 21.6)^f^	0.058	6.7 ± 3.6	5.2 (2.2, 8.1)	0.515	0.028	0.216
ALT (U/L)	57.0 (33.8, 105.5)	50.0 (37.0, 130.0)	0.543	52.4 ± 19.6	47.0 (29.0, 82.0)	0.859	0.433	0.502
AST (U/L)	64.0 (38.0, 86.5)	73.0 (44.0, 134.0)	0.375	59.5 ± 22.1	58.0 (38.8, 90.5)	0.441	0.505	0.333
ALP (U/L)	270.5 (209.8, 438.3)	325.0 ± 176.1	0.639	206.3 ± 47.5	258.8 ± 130.3	0.477	0.006	0.238
GGT (U/L)	110.5 (38.5, 195.8)	105.0 (60.0, 220.0)	0.244	102.5 (34.8, 187.5)	57.0 (23.3, 217.8)	0.953	0.875	0.404
Tbi (µmol/L)	36.6 ± 64.9	19.9 (5.4, 56.9)	0.000	11.8 (6.1, 16.4)	7.2 (4.3, 10.7)	0.308	0.480	0.015
Dbi (µmol/L)	21.4 ± 46.4	3.1 (1.2, 23.8)	0.244	2.3 ± 5.9	1.9 (0.0, 4.3)	0.046	0.015	0.072

*TBA, total bile acids; ALT, alanine aminotransferase; AST, aspartate aminotransferase; ALP, alkaline phosphatase; GGT, γ-glutamyltransferase; Tbi, total bilirubin; Dbi, direct bilirubin.*

*^a^* *Comparison of hepatic function before anastomosis.*

*^b^* *Comparison of hepatic function after anastomosis.*

*^c^* *Comparison of hepatic function at 2 weeks before and after anastomosis.*

*^d^* *Comparison of hepatic function at 4 weeks before and after anastomosis.*

*^e^* *Mean ± standard deviation (all such values).*

*^f^* *Median (interquartile range) (all such values).*

## Discussion

One of the most important management strategies in pediatric IF cases is providing sufficient fluids, electrolytes, and nutrients to maintain normal growth (1–3). Except for receiving parenteral and/or enteral nutrition support therapies passively, the human body can increase its absorptive capacity automatically by postresection intestinal adaptation. The process of intestinal adaptation that occurs following extensive intestinal resection has been described as a natural compensatory process where the remaining bowel undergoes substantial structural and functional changes that increase its absorptive capacity (1, 19). Owing to the small size of the SBS population and the invasive nature of many of the procedures required to assess the structural and functional adaptation, animal studies investigating postresection intestinal adaptation are relatively richer than human studies (19). In animal studies, postresection structural adaptations include bowel lengthening and thickening, an increase in the villus height and crypt depth, and functional changes including increased nutrient transporter expression, accelerated crypt cell differentiation, and a slower transit time (19–21). However, specific figures regarding postresection adaptive intestinal elongation have seldom been reported. In our study, the postresection average growth (cm/month) in the lengths of the proximal and distal intestine was calculated as growth in the intestinal length (cm)/the duration (month) from enterostomy to anastomosis. Feldman et al. reported that luminal nutrients enhance postresection adaptation (22); furthermore, increased nutrient complexity is associated with greater adaptation (23). In this study, there was greater growth in the proximal intestinal length which is exposed to more luminal nutrients and increased nutrient complexity that act as potent stimuli for intestinal growth.

IFALD is considered to be multifactorial and is the most frequent, even life-threatening, complication of pediatric IF resulting from long-term PN (6). IFALD can be defined as hepatobiliary dysfunction as a consequence of medical and surgical management strategies for intestinal failure which can be stabilized or reversed with promotion of intestinal adaptation (9, 24). The colon plays an important role in intestinal adaptation in patients with SBS (25), as it may induce changes that allow the colonic mucosa to enhance its capacity for water and electrolyte absorption as well as modifications that allow absorption of nutrients when undigested nutrients are exposed to the colon (26, 27). In pediatric type I SBS, once intestinal continuity is restored through an anastomosis after ileostomy or jejunostomy, the colon is exposed to undigested nutrients, and trophic hormones such as enteroglucagon may be stimulated which contribute to intestinal adaptation; thus, the process of IFALD may be stabilized or reversed (9, 24, 25). IFALD has a multifactorial etiology, and no single factor has been implicated as the main culprit (9). A series of pathophysiological changes including a paucity of oral and enteral nutrition, impaired enterohepatic circulation of bile acids, small intestinal bacterial overgrowth (SIBO) and translocation of toxin-producing bacteria to the portal circulation, and an abnormal transit time may occur after ileostomy or jejunostomy which may lead to liver inflammation with further progression of IFALD (28). The lack of enteral feeding impairs the enterohepatic circulation and bile acid secretion/absorption leading to mucosal atrophy and an increased risk of bacterial translocation (9). After closure of the stoma, the intestinal continuity is restored and enterohepatic circulation of bile acids is remodeled which can improve enteral feed tolerance and bile acid secretion/absorption and reduce bacterial overgrowth. Thus, some of the mechanisms relevant to the pathogenesis of IFALD can potentially be modified which may, theoretically, have a positive effect on the prevention or resolution of IFALD (17, 29, 30). The results of our study showed that the incidence of IFALD and serum levels of TBA, ALP, Tbi, and Dbi decreased significantly after restoration of intestinal continuity in 35 pediatric IF patients with type I SBS; these results are in accordance with those of previous studies.

However, our study has some limitations. The retrospective data collection did not allow us to introduce accurate variables related to the initial clinical status, and the baseline characteristics of patients are difficult to standardize. The second limitation is that this was a single-center study with a small sample size which may weaken the statistical power and the validity of our conclusions. Although most IFALD cases are preventable and reversible, IFALD is still a potential cause of end-stage liver disease and remains one of the major indications for intestinal or combined liver and small bowel transplantation (6). Therefore, more multi-center RCT studies with high quality, large sample, and adequate follow-up are required for further verification.

## Conclusion

In pediatric type I SBS with IFALD, the restoration of intestinal continuity may alleviate liver injury. Additionally, there was an intestinal compensatory effect on the growth in intestinal length after resection, and the better results were observed in neonates with respect to the growth in intestinal length among the pediatric type I SBS population.

## Data Availability

The original contributions presented in the study are included in the article/Supplementary Material, further inquiries can be directed to the corresponding author/s.
